# Factors Affecting Adherence to Hemodialysis Therapy Among Patients With End-Stage Renal Disease Attending In-Center Hemodialysis in Al-Ahsa Region, Saudi Arabia

**DOI:** 10.7759/cureus.46701

**Published:** 2023-10-09

**Authors:** Mahdi A Alhamad, Mohammed Y Almulhim, Abdullah A Alburayh, Razan A Alsaad, Arwa M Alhajji, Jawad S Alnajjar, Sawsan S Alhashem, Ghassan Salah, Muthana Al Sahlawi

**Affiliations:** 1 Nephrology, King Faisal University, Al-Ahsa, SAU; 2 Internal Medicine, College of Medicine, King Faisal University, Al-Ahsa, SAU; 3 Nephrology, King Fahad General Hospital, Al-Ahsa, SAU

**Keywords:** adherence to therapy, spss (statistical package for the social sciences), peritoneal dialysis (pd), hd ( hemodialysis ), renal replacement therapy (rrt), end stage renal disease (esrd), ckd(chronic kidney disease)

## Abstract

Background

Chronic kidney disease (CKD) and end-stage renal disease (ESRD) are global health concerns, with ESRD requiring renal replacement therapy (RRT). Hemodialysis is a prevalent modality for RRT. However, access to hemodialysis is challenging for rural patients due to geographical barriers and limited nephrology services. This research aims to identify factors influencing adherence to hemodialysis sessions among rural ESRD patients, addressing travel, healthcare infrastructure, and socioeconomic factors.

Materials and methods

A cross-sectional study of 154 participants was conducted from July 06 to September 10, 2023 at Al-Jaber Dialysis Center in Al-Ahsa, Saudi Arabia. It included adult CKD patients on hemodialysis who were interviewed to assess factors influencing hemodialysis adherence using a structured questionnaire.

Results

Our study assessed hemodialysis adherence in 154 patients in Al-Ahsa, Saudi Arabia. Gender distribution was nearly equal (male = 54.5%), with the majority aged 41-60, married, and residing in downtown areas. Hypertension (43.9%) and diabetes (32.3%) were the prevalent comorbidities. Most patients received thrice-weekly dialysis (96.15%), with family cars as the primary transportation mode (55.2%). Hypertension (43.3%) and diabetic nephropathy (40.9%) were the leading causes of CKD. Approximately 26% missed dialysis, with health issues and transportation difficulties being common reasons. Notably, adherence correlated with female gender, lower education, and family car transportation mode. Social support significantly influenced adherence, highlighting its importance in maintaining hemodialysis adherence.

Conclusion

Our study identified various sociodemographic and dialysis-related factors influencing adherence among hemodialysis patients in the Al-Ahsa region, Saudi Arabia. Notably, factors such as gender, education level, and transportation means significantly influenced adherence. Adequate family and social support were associated with better adherence. These findings highlight the importance of tailored interventions addressing these factors to enhance hemodialysis adherence and ultimately improve patient outcomes in this population.

## Introduction

Chronic kidney disease (CKD) is a non-communicable disease characterized by structural or functional abnormalities of the kidneys lasting more than three months and causing adverse effects. CKD ranges from Stage 1 (mild) to Stage 5, which is an end-stage renal disease (ESRD) and requires dialysis or kidney transplant surgery [1]. The prevalence of ESRD is increasing worldwide [2]. Current data suggest that CKD affects 9.1%-13.4% of the world's population and that its prevalence is increasing worldwide, in part due to risk factors such as obesity and diabetes [1]. In Saudi Arabia, CKD has been recognized as a major health problem in recent decades due to the increasing incidence and prevalence of ESRD among the Saudi population [3].

The occurrence of ESRD leads rapidly to death unless renal replacement therapy (RRT) is initiated [4]. There are different modalities for RRT, including hemodialysis, peritoneal dialysis (PD), and kidney transplantation. Hemodialysis is one of the most popular and effective treatments for ESRD patients [5]. There has been a 35% increase in the number of dialysis patients worldwide [6]. In Saudi Arabia, hemodialysis patients' average net annual growth is 6% [7]. In 2021, there were over 20,000 patients on dialysis and 9,810 patients undergoing follow-up after kidney transplantation. Total RRT prevalence in Saudi Arabia is estimated at 294.3 per million people [8].

In developed areas, there are usually sufficient dialysis centers, so patients are assigned to a dialysis facility close to their residence. The opposite is true in underdeveloped areas [9]. In Saudi Arabia, there are 278 hemodialysis centers with 8,165 dialysis machines to meet the growing demand [2].

Patients from rural areas may not be able to access their preferred option of RRT due to the difficulty of traveling far to clinical appointments and the high expense of offloading [10]. Also, remote inhabitants must travel long distances to access specialized medical care and experience additional system-level barriers to healthcare delivery, such as lower income, geographical isolation, and imbalances in physicians' supply [11]. In addition, access to all forms of dialysis and kidney transplantation for rural patients can be challenging due to late referral and limited local availability of specialist nephrology services [12]. Furthermore, non-attendance may be associated with an increasing frequency of emergency department utilization and hospitalization. For these reasons, we aim to determine factors affecting adherence to hemodialysis sessions among hemodialysis patients using an interview-based study.

## Materials and methods

Study design, setting, and participants

A descriptive cross-sectional study was carried out at Al-Jaber Dialysis Center in Al-Ahsa region, Saudi Arabia during the period from July 6 to September 10, 2023. The aim of this study was to evaluate the adherence and attendance pattern toward hemodialysis sessions among CKD patients. It targeted Saudi individuals who were 18 years and above and undergoing hemodialysis treatment at Al-Jaber Dialysis Center in Al-Ahsa. The study was approved by the Ethics Committee of King Fahad Hospital in Al-Ahsa (49-EP-2023, date of approval July 6, 2023). The required sample size was 132 using the formula n=Z2 pq/E2, where the margin of error (E) equals 0.05. The confidence level (Za/2) was 95%, which equals 1.96. The expected proportion (p) of adults equals 0.5; the actual sample size was 154 randomly selected patients. A convenient sampling technique was employed to collect the data.

Questionnaire development and data collection

Data was collected through patients’ interviews during their hemodialysis sessions using a structured questionnaire. Prior to collecting data from the participants, informed consent was obtained. The study utilized a questionnaire that was adopted from a previous study with some modifications [13]. Face and content validity techniques were used to create and validate the questionnaire. Face validity was achieved by administering the draft questionnaire to a few patients who met the inclusion criteria at Al-Jaber Hemodialysis Center in order to determine whether the response appeared meaningful, well-designed, and/or a good measure of the construct to an innocent participant. The questionnaire was further improved and altered using the data gathered from this exercise. Four independent researchers from the field of Nephrology evaluated the questionnaire's appropriateness, clarity, coverage, and relevance to the study as part of the content validity process. The reliability of the questionnaire was calculated using Cronbach’s alpha test, and the result showed a Cronbach’s alpha value of 0.837, indicating that the questionnaire was highly reliable.

Patients receiving hemodialysis participated in interviews to complete the questionnaire. The questionnaire consists of two sections. The first section focused on personal and demographical data which include age, gender, marital status, region, education level, employment status, distance to the nearest dialysis center, transportation method to the dialysis center, comorbidity, the cause of kidney disease that led to the need for being on dialysis and previous kidney transplantation. In the second section, questions were asked regarding the duration of dialysis, the number of sessions per week that patients' doctors prescribed, missed sessions over the previous three months, the recommended number of hours per session, requests for early dialysis session termination, family and social support, visits from social workers, whether the doctor had discussed the significance of adherence to dialysis, and whether or not the patient thought that dialysis was important.

Statistical analysis 

Descriptive statistics were used to summarize the responses. Frequency distributions and percentages of the socio-demographic characteristics and other categorical variables were calculated and tabulated. Testing the association was by the Chi-Square and Fisher's exact tests. Qualitative variables were represented as percentages, and numbers and were shown in the figures. All statistical analyses were conducted using Statistical Package for the Social Sciences (SPSS) version 26.0 (IBM Corp., Armonk, NY, USA), and a p-value of less than 0.05 was considered statistically significant.

## Results

Table 1 shows a total of 154 Saudi participants were included in the study and represents their sociodemographics. Regarding gender, 45.5% were female, and 54.5% were male. Regarding age, <20 years (8.4%), 21-40 years (20.8%), 41-60 years (44.8%), and >60 years (26.0%). Marital status showed 22.7% single, 57.8% married, 5.8% divorced, and 13.6% widowed individuals. Region-wise, 68.8% were from downtown, 27.9% from rural areas, and 3.2% from immigration areas. Educational levels ranged from no education (31.8%) to post-graduation (0.6%). Employment status included 19.5% employed, 20.1% retired, and 60.4% unemployed individuals.

**Table 1:** Sociodemographic and other parameters of all patients assessed for dialysis Immigration area: A village or urban settlement created to settle Bedouin tribes.

		Frequency (n=154)	Percent
Gender	Female	70	45.5
	Male	84	54.5
Age	< 20 Years	13	8.4
	21-40 Years	32	20.8
	41-60 Years	69	44.8
	> 60 Years	40	26.0
Marital Status	Single	35	22.7
	Married	89	57.8
	Divorced	9	5.8
	Widow	21	13.6
Region	Downtown	106	68.8
	Rural area	43	27.9
	Immigration areas	5	3.2
Educational Level	No Education	49	31.8
	Primary	27	17.5
	Secondary	32	20.8
	Intermediate	23	14.9
	Bachelors	12	7.8
	Masters	1	.6
	Diploma	9	5.8
	Post Graduation	1	.6
Employment	Employed	30	19.5
	Retired	31	20.1
	Unemployed	93	60.4

Table 2 shows dialysis-related parameters. The distance to the nearest dialysis center varied, with 42.9% within 10 km, 36.4% within 10-20 km, and 21.0% beyond. Transportation methods included family cars (55.2%), self-driven cars (27.3%), and paid service (17.5%). Dialysis duration showed 13.6% for <1 year, 22.7% for one to two years, and 63.6% for >2 years. The majority (96.1%) were prescribed three sessions of dialysis per week, while a few had two (1.3%) or four (2.6%) sessions.

**Table 2:** Dialysis parameters of patients

		Frequency (n=154)	Percent
Distance to Nearest Dialysis Center	< 10 km	66	42.9
	10-20 km	56	36.4
	20-30 km	20	13.0
	> 30 km	12	7.8
Method of Transportation to Dialysis Center	Car (by Family member)	85	55.2
	Car (by Myself)	42	27.3
	Car (by Paid service)	27	17.5
Periods on Dialysis	< 1 Year	21	13.6
	1-2 years	35	22.7
	> 2 years	98	63.6
Prescribed dialysis Sessions/week	2	2	1.3
	3	148	96.1
	4	4	2.6

**Figure 1:**
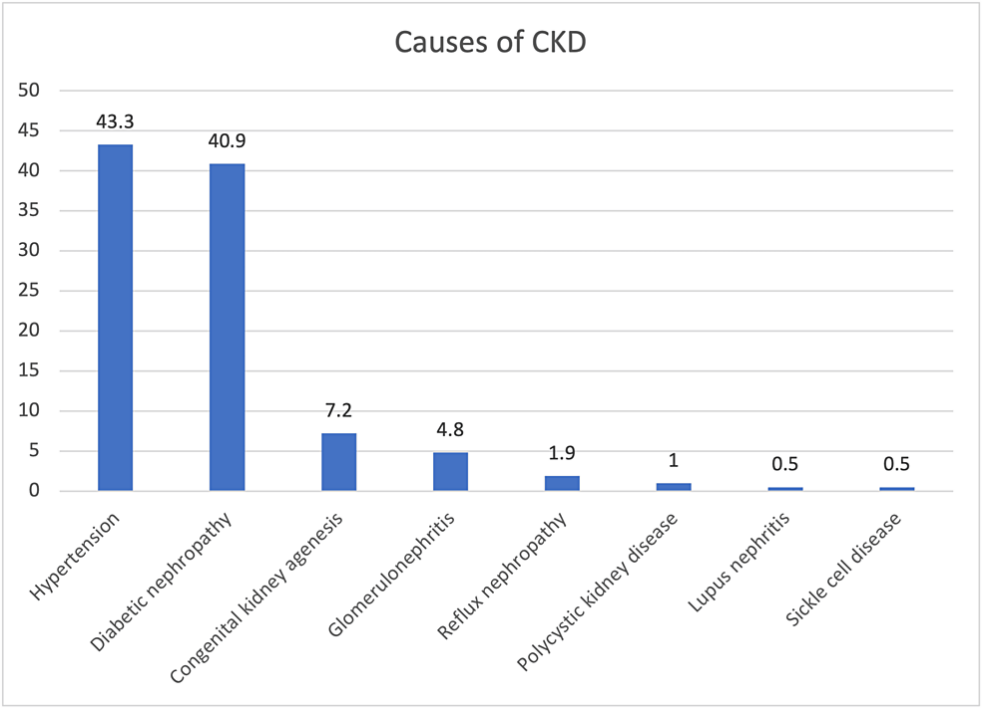
Causes of CKD which leads to dialysis CKD - chronic kidney disease

The primary causes of CKD, which leads to ESRD Dialysis among patients were hypertension (43.3%) and diabetic nephropathy (40.9%). Less common causes included congenital kidney agenesis (7.2%), glomerulonephritis (4.8%), reflux nephropathy (1.9%), polycystic kidney disease (1%), lupus nephritis (0.5%), and sickle cell disease (0.5%) (Figure 1).

Table 3 shows that 26.0% missed dialysis in the last three months, with 47.5% missing one session, 17.5% missing two, and 35.0% missing three or more. About 16.2% requested early termination, mostly one session (44.0%). The majority (89.6%) had family and social support for adherence. Most patients (79.2%) had doctors discussing the importance of adherence, with 61.0% prescribed four-hour sessions. Overall, 91.6% recognized the importance of adhering to hemodialysis sessions.

**Table 3:** Assessment of dialysis adherence

		Frequency (n=154)	Percent
Missed dialysis in Last 3 Months	No	114	74.0
	Yes	40	26.0
No. of sessions missed per month	1	19	47.5
	2	7	17.5
	3 or more	14	35.0
Requested for termination of Dialysis over the Last 3 Months	Yes	25	16.2
No. of sessions requested to terminate Early	1	11	44.0
	2	9	36.0
	3 or more	5	20.0
Enough Family & Social Support to adhere to hemodialysis	Yes	138	89.6
Your doctor discusses the importance of dialysis adherence	Yes	122	79.2
No. of hours per session prescribed by your doctor	3.5h	48	31.2
	3h	12	7.8
	4h	94	61.0
Adherence to hemodialysis sessions is important	Yes	141	91.6

**Figure 2:**
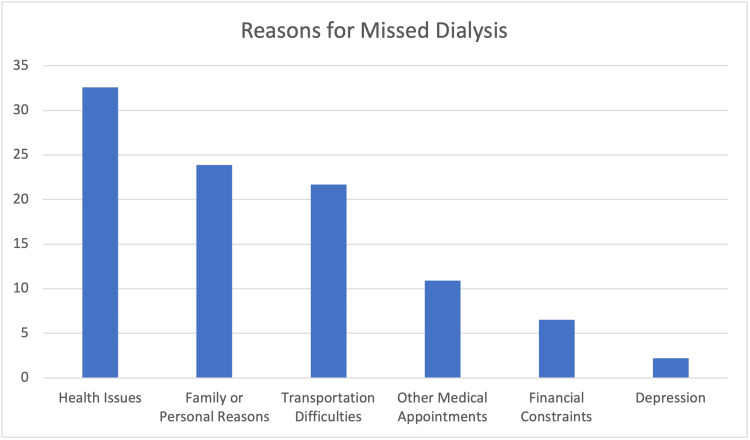
Reasons for missed dialysis

Patients missed dialysis due to various reasons: health issues (32.6%), family or personal reasons (23.9%), transportation difficulties (21.7%), other medical appointments (10.9%), financial constraints (6.5%), and depression (2.2%) (Figure 2).

Table 4 shows the various sociodemographic factors associated with dialysis adherence. Good adherence was defined as patients who did not miss any dialysis sessions over the last three months. Poor adherence was defined as patients who missed one or more hemodialysis sessions over the last three months without any reason. Good adherence was more likely among females (p=0.008), those with no or lower than bachelor's level (p=0.007), and those using family cars for transportation (p<0.001). Age, marital status, region, employment status, and distance to the dialysis center did not significantly affect adherence (p>0.05). These findings suggest gender, education, and transportation methods play significant roles in adherence to hemodialysis treatment.

**Table 4:** Different sociodemographic features associated with dialysis adherence

		Adherence to Hemodialysis Treatment		Sig. Value
		Poor Adherence	Good Adherence	
Age	< 20 Years	3	10	0.084
	21-40 Years	12	20	
	41-60 Years	20	49	
	> 60 Years	5	35	
Gender	Female	11	59	0.008
	Male	29	55	
Marital Status	Single	10	25	0.685
	Married	25	64	
	Divorced	1	8	
	Widow	4	17	
Region	Downtown	26	80	0.701
	Immigration areas	2	3	
	Rural area	12	31	
Educational Level	No Education	7	42	0.007
	Primary	14	13	
	Secondary	7	25	
	Intermediate	5	18	
	Bachelors	5	7	
	Masters	1	0	
	Diploma	1	8	
	Post Graduation	0	1	
Employment	Employed	12	18	0.095
	Retired	5	26	
	Unemployed	23	70	
Distance to Nearest Dialysis Center	< 10 km	16	50	0.843
	10-20 km	14	42	
	20-30 km	6	14	
	> 30 km	4	8	
Transportation Method	Car (by family member)	15	70	<0.001
	Car (by patient)	21	21	
	Car (by paid service)	4	23	

Table 5 shows the various dialysis-related factors were examined for their association with adherence. Notably, good adherence was linked to having enough family and social support (p=0.032). Other factors such as the period on dialysis, prescribed sessions per week, interactions with social workers, doctor discussions, hours per session, and personal opinions on the importance of adherence did not significantly impact adherence (p>0.05). This underscores the significance of social support in maintaining dialysis adherence.

**Table 5:** Different dialysis-related features associated with dialysis adherence

		Adherence to Hemodialysis Treatment		Sig. Value
		Poor Adherence	Good Adherence	
Period on Dialysis	< 1 Year	5	16	0.228
	1-2 years	13	22	
	> 2 years	22	76	
Prescribed dialysis Sessions/week	2	0	2	0.601
	3	40	108	
	4	0	4	
Enough Family & Social Support	Yes	32	106	0.032
Seen a dialysis unit social worker over the last month	Yes	29	82	0.945
Your doctor discusses the Importance of Dialysis	Yes	30	92	0.444
No. of hours/session prescribed by your doctor	3.5h	17	31	0.106
	3h	1	11	
	4h	22	72	
Adherence with Dialysis is important in Your Opinion	Yes	35	106	0.283

## Discussion

Adherence to hemodialysis therapy is critical for the management of ESRD but often poses a significant challenge for patients. Our study focused on understanding factors influencing hemodialysis adherence. We uncovered an intricate interplay of demographic, clinical, and social factors that profoundly affect adherence, ultimately shaping patient outcomes and quality of life. This research sheds light on the complexity of managing CKD and highlights the importance of holistic care in ESRD treatment.

Demographic characteristics play a crucial role in understanding the patient population and tailoring interventions. Our study showed a near gender parity among patients, with 45.5% being female and 54.5% male. This distribution aligns with the global trend of ESRD affecting the male gender more as compared to the female gender, emphasizing the importance of gender-based interventions [14,15]. Age distribution revealed that the majority of patients fell within the 41-60 years age group (44.8%), indicating that this age group often faces multiple challenges, including comorbid conditions, family responsibilities, and the need for additional support. Alkatheri et al. show that older significantly increase their adherence level to hemodialysis in contrast with our findings in which age is not significantly associated with adherence [16]. Marital status showed that a significant portion of patients were married (57.8%), suggesting potential family support systems [17]. However, 13.6% were widowed, possibly facing emotional and logistical challenges that could affect adherence. These findings highlight the importance of involving family members in the care and decision-making process for patients, particularly those without spousal support.

Educational levels varied widely, with 31.8% having no formal education. Lower educational attainment was associated with better adherence, which contrasts with some previous studies [18,19]. This unexpected result may indicate that patients with lower education levels in this context have more robust support systems or better communication with healthcare providers. Nevertheless, tailored educational interventions remain crucial, focusing on improving health literacy and self-management skills [20]. Employment status revealed that a significant proportion (60.4%) of patients were unemployed. Unemployment can impact not only financial stability but also psychological well-being, potentially affecting adherence. Addressing the psychosocial aspects of unemployment is vital in comprehensive patient care.

Hypertension (43.9%) and diabetes (32.3%) are prevalent comorbidities in ESRD, aligning with established links to CKD. Managing these conditions is pivotal in preventing kidney disease progression and enhancing treatment outcomes [21]. Hypertension (43.3%) and diabetic nephropathy (40.9%) were considered as primary causes leading to ESRD. It emphasizes the need for effective prevention and management of hypertension and diabetes to reduce ESRD incidence [22]. Most patients adhered to the standard prescription of three weekly dialysis sessions (96.1%). However, 26.0% reported missed sessions, mainly due to health issues (32.6%), personal reasons (23.9%), and transportation challenges (21.7%) [23]. Notably, patients using family cars (55.2%) demonstrated better adherence, emphasizing the importance of transportation methods and family support in treatment compliance [24].

Social support emerged as a critical factor influencing adherence. The majority of patients (89.6%) reported having family and social support for adherence. This finding emphasizes the need to involve family members and caregivers in the patient's care plan. Family support can provide emotional assistance and help address logistical challenges, ultimately promoting better adherence [25,26]. Moreover, most patients (79.2%) reported that doctors discussed the importance of adherence with them. These discussions are pivotal in reinforcing the significance of adhering to hemodialysis sessions. Healthcare providers must continue these conversations, addressing patient concerns and providing clear information about the benefits of adherence.

Various sociodemographic and dialysis-related factors are associated with adherence. Notably, good adherence was more likely among females. This is in contrast to previous studies where the male gender shows good adherence to dialysis [27]. This could be attributed to diverse factors such as sociodemographic variations, cultural influences, and regional healthcare access. Individual motivations and social support networks may also play pivotal roles. It's essential to recognize that adherence is a complex, multifaceted behavior influenced by various determinants. So, gender-related adherence patterns may differ across distinct patient populations and settings.

Adherence to hemodialysis appears unaffected by sociodemographic factors. Family and social support are the pivotal factors influencing treatment compliance.

Study limitations

There are several limitations of the study which include a relatively small sample size, which may limit the generalizability of findings. Data collected through interviews may be subject to recall bias. The study's cross-sectional design prevents the establishment of causal relationships. Additionally, the study's focus on a single center in a specific region may not represent the diversity of adherence patterns in all hemodialysis patients across Saudi Arabia.

## Conclusions

Our study identified various sociodemographic and dialysis-related factors influencing adherence among hemodialysis patients in the Al-Ahsa region, Saudi Arabia. Notably, factors such as gender, education level, and transportation means significantly influenced adherence. Adequate family and social support were associated with better adherence. These findings highlight the importance of tailored interventions addressing these factors to enhance hemodialysis adherence and ultimately improve patient outcomes in this population.
